# Country-Level Structural Stigma, School-Based and Adulthood Victimization, and Life Satisfaction Among Sexual Minority Adults: A Life Course Approach

**DOI:** 10.1007/s10964-020-01340-9

**Published:** 2020-11-16

**Authors:** Arjan van der Star, John E. Pachankis, Richard Bränström

**Affiliations:** 1 Division of Psychology, Department of Clinical Neuroscience, Karolinska Institutet, Stockholm, Sweden; 2 Department of Social and Behavioral Sciences, Yale University School of Public Health, New Haven, CT USA

**Keywords:** Sexual minorities, Disclosure, Victimization, Stigma, Mental health

## Abstract

Country-level structural stigma, defined as prejudiced population attitudes and discriminatory legislation and policies, has been suggested to compromise the wellbeing of sexual minority adults. This study explores whether and how structural stigma might be associated with sexual minorities’ school-based and adulthood experiences of victimization and adulthood life satisfaction. Using a sample of 55,263 sexual minority individuals (22% female; 53% 18–29 years old; 85% lesbian/gay, 15% bisexual) living across 28 European countries and a country-level index of structural stigma, results show that sexual minorities, especially men, reported school bullying in both higher- and lower-stigma countries. Higher rates of school bullying were found among sexual minorities living in higher-stigma countries when open about their identity at school. Past exposure to school bullying was associated with lower adulthood life satisfaction, an association partially explained by an increased risk of adulthood victimization. These findings suggest that sexual minorities living in higher-stigma countries might benefit from not being open about their sexual identity at school, despite previously established mental health costs of identity concealment, because of the reduced risk of school bullying and adverse adulthood experiences. These results provide one of the first indications that structural stigma is associated with sexual minority adults’ wellbeing through both contemporaneous and historical experiences of victimization.

## Introduction

According to life course theory and research, exposure to country-level structural stigma (i.e., prejudiced population attitudes and discriminatory legislation and policies) toward sexual minorities (e.g., those who identify as gay, lesbian, or bisexual) during childhood may not only compromise sexual minority youth’s wellbeing through school-based experiences, but it may also yield negative consequences throughout the life course. Together with the negative sequelae of adulthood exposure to structural stigma, such as victimization, persisting effects of historical exposure may hypothetically accumulate throughout sexual minorities’ lives. Despite the fact that some studies have examined correlates of structural stigma exposure during both childhood and adulthood, they have done so separately and none has yet investigated this association and its potential mediators using a life course approach. The present study addresses this gap by examining how experiences during childhood, such as school bullying and sexual identity openness at school, and a subsequent risk for adulthood victimization may explain how country-level structural stigma jeopardizes sexual minorities’ life satisfaction in adulthood.

Structural stigma has been identified as an important risk factor for poor mental health among sexual minorities (Hatzenbuehler 2016). Structural stigma has been previously defined and is typically measured as negative population attitudes, cultural norms, discriminatory legislation and policies, and unequal rights that hamper the wellbeing and opportunities of stigmatized groups (Hatzenbuehler 2016). Across nations worldwide, the structural climate surrounding sexual minorities varies widely (Flores 2019). In Europe, societal attitudes toward sexaul minorities, measured as the percentage of the population agreeing that gays and lesbians should be free to live their lives as they wish, ranged from about 30% in Ukraine and Russia to around 90% in the Netherlands, Sweden, and Denmark in 2010, which also correlate with a legal index on human rights protections or violations in those countries (Bränström and Van der Star 2013).

Adulthood exposure to structural forms of stigma has been consistently associated with poorer mental health outcomes among sexual minority adults, including lower life satisfaction (Pachankis and Bränström 2018) and higher psychological distress (Hatzenbuehler et al. 2018). Beside these bivariate associations between structural stigma and poor mental health, structural stigma may also indirectly compromise mental health. A growing number of studies has started to identify possible psychosocial mechanisms through which structural stigma might reduce adulthood mental health, such as through victimization, as a form of stigma at the interpersonal level, and sexual identity concealment, as a stigma-related factor at the individual level (e.g., Pachankis and Bränström 2018). However, these studies describe associations with contemporaneous exposure to structural stigma and, so far, potential correlates of historical exposure to structural forms of stigma and sexual minorities’ wellbeing remain relatively unknown.

Structural stigma exposure during childhood may have an immediate effect on sexual minorities’ mental health by inducing adverse childhood experiences, but historical exposure to structural stigma may also lead to detrimental consequences for sexual minorities’ adulthood wellbeing and an increased vulnerability to adulthood victimization. During childhood, structural stigma might promote adverse experiences among sexual minorities, including school bullying (Saewyc et al. 2014), yet few studies have examined this possibility. Although homophobic school environments have been linked to higher rates of stigma-based bullying among sexual minority youth (e.g., Kull et al. 2016), less is known about how structurally stigmatizing national climates surrounding sexual minority youth may be associated with stigma-based school bullying.

During adulthood, historical exposure to structural stigma may lead to detrimental consequences for sexual minorities’ wellbeing through an increased vulnerability to adulthood victimization. According to theory and research on life course stressors, increased vulnerability to adulthood victimization might stem from cascading effects throughout the lifespan. Indeed, stigma-based victimization in childhood may cause proliferation of secondary stressors, including stigma-based victimization in adulthood (Gee et al. 2012). For instance, childhood bullying may cause proliferation of secondary stressors (Gee et al. 2012), through lower self-confidence, feelings of identity-related shame, and unassertiveness in social interactions later in life (Slavich et al. 2010), which in turn could predispose individuals to future harassment and victimization (Robinson et al. 2013).

Although few moderators of the above associations between structural stigma and childhood and adulthood stigma-based victimization have been examined, concealment of sexual identity might represent an important means for sexual minority youth to navigate hostile environments and potentially avoid victimization, particularly in structurally unsupportive climates (Pachankis and Bränström 2018). Specifically, the association between structural stigma and school bullying may vary as a function of sexual identity openness, as concealment of sexual identity may dampen this association (Pachankis and Bränström 2018). With sexual minority boys reportedly being at a higher risk for school bullying than sexual minority girls at least in lower-stigma countries, such as the Netherlands (Collier et al. 2013a), but not necessarily in higher-stigma settings, such as the US (Kosciw et al. 2014), sexual minority boys and girls may differ in their ability to mitigate the risk of school bullying across structural environments. For instance, concealment of sexual orientation might be a less viable tool for sexual minority boys, compared to sexual minority girls, in navigating the risk of school bullying across structural contexts. In fact, sexual identity-based school bullying is not only targeted at those who publicly self-identify and disclose sexual minority identities, but also at those perceived as such (D’Augelli et al. 2002), particularly among sexual minority boys (Van Beusekom et al. 2020). In sum, while country-level structural stigma may be associated with increased risk of school bullying among sexual minorities, the association between country-level structural stigma and school bullying may vary as a function of sexual identity openness at school and may do so more strongly among sexual minority girls, who might be less targeted than sexual minority boys and have more options for concealing their current or nascent identities.

## Current Study

In order to examine the potential life course influences of early exposure to structural stigma, the current study used data from 28 European countries to examine whether country-level structural stigma exposure during childhood is associated with sexual minorities’ adulthood life satisfaction through school-based experiences, namely school bullying and identity openness at school, directly but also indirectly through a subsequent higher risk for adulthood victimization. To investigate these life course correlates of structural stigma, this study tested a set of four hypotheses. First, sexual minorities living in European countries with higher levels of structural stigma will be more likely to report school bullying than sexual minorities living in countries with lower levels (Hypothesis 1). Second, the association between country-level structural stigma and school bullying will vary based on identity openness at school, but more strongly so among sexual minority women (Hypothesis 2). Third, school bullying will be associated with reduced adulthood life satisfaction, both directly and indirectly through a subsequent higher risk for adulthood victimization (Hypothesis 3). Fourth, the association between country-level structural stigma and adulthood life satisfaction will be mediated by a higher risk for school bullying and subsequent adulthood victimization among sexual minorities who were more open about their identity at school (Hypothesis 4).

## Methods

### Participants

This study used cross-sectional data from 93,079 respondents who were 18 years or older; living in one of the 28 EU member states; self-identifying as lesbian, gay, bisexual, or transgender (LGBT); and participating in the EU-LGBT survey. The survey was conducted by the European Union Agency for Fundamental Rights in 2012 (European Union Agency for Fundamental Rights 2014) and participants were recruited through social networking websites, mailing lists, press releases, LGBT organizations, and dating websites. The number of respondents varied by country (e.g., Germany [*n* = 20,271] to Luxembourg [*n* = 318]). Further details about the data collection and translation procedures have been published elsewhere (European Union Agency for Fundamental Rights 2014). As the skip logic of the online survey was set up so that those identifying as transgender were not asked about bullying due to their sexual identity, gender minorities were excluded from the study (*n* = 6771; 7.3%). Given the life course perspective of this study, the sample was further restricted by consecutively excluding those who had not spent their school years in their current country of residence (*n* = 7763; 8.3%), lived outside the EU (*n* = 328; 0.4%), and had missing data on any of the key study variables (*n* = 22,954; 24.6%). A total of 55,263 sexual minority individuals were included.

Regarding the missingness of data, differences were tested between those included in the study and those who were excluded from the study on the basis of having missing data on any of the key study variables. The included sample was more likely to be male, to be younger, to report an ethnic minority status, to have not attended university, and to report lower household income than the excluded group with missing data. Compared with this excluded group, the included sample was also more likely to live in higher-stigma countries (M_*diff*_: 0.18, *p* < 0.001), to be open at school (AOR: 5.18, *p* < 0.001), to be victimized in adulthood (AOR: 1.14, *p* < 0.001), and to report lower adulthood life satisfaction (M_*diff*_: −0.14, *p* < 0.001).

### Individual-Level Measures

#### Sexual identity

Respondents were asked which of the following categories best described them with regard to their sexual minority identity, namely “lesbian”, “gay”, “bisexual”, or “other”.

#### School bullying

Identity-targeted school bullying was measured with a question tailored to the participant’s reported sexual identity: “During your schooling before the age of 18, did you experience negative comments or conduct at school because of you being lesbian/gay/bisexual?” on a scale from “never”, “rarely”, “often”, to “always” or “does not apply”. Based on the variable distribution and uneven spacing between ordinal categories, responses were dichotomized into either never and rarely bullied at school (= *Not bullied*) or often and always bullied at school because of being lesbian, gay, or bisexual (LGB) (= *Bullied*) to facilitate the interpretation of the analyses.

#### Identity openness at school

The level of openness about the respondent’s self-reported sexual identity at school was measured with the question “During your schooling before the age of 18, did you openly talk about you being LGB at school?” Responses were measured on a scale from “never”, “rarely”, “often”, to “always” or “does not apply”. Based on the variable distribution and uneven spacing between ordinal categories, identity openness at school was then dichotomized to contain never and rarely open (= *Low openness*) versus often and always open (= *High openness*) to facilitate the interpretation of the analyses.

#### Adulthood victimization

The frequency of experienced victimization in adulthood was measured with the question “How many times did someone physically/sexually attack or threaten you with violence in the last 12 months in the European Union/in this country?” Based on the skewed distribution of the variable, response options were dichotomized to contain no experiences of victimization during the past 12 months (= *Not victimized*) versus any exposure to past-year victimization (= *Victimized*).

#### Adulthood life satisfaction

Life satisfaction in adulthood was measured with the question “All things considered, how satisfied would you say you are with your life these days?” with response alternatives ranging from 1 = *Very dissatisfied* to 10 = *Very satisfied*. This item demonstrates high within-person stability and high validity across time, societal conditionals, and cultures and is consistently associated with quality of life and various mental health outcomes (e.g., Diener et al. 2013).

#### Other socio-demographic variables

Socio-demographic covariates included in the analyses were age and household income based on participant’s self-reported monthly net household income, per preset country-specific population quartiles. These factors were used as individual-level covariates given that life satisfaction is negatively correlated with both younger age (Westerhof and Keyes 2010) and low income (Sacks et al. 2010). To characterize the demographic makeup of the sample, we examined other socio-demographic variables: gender, ethnic minority status, level of educational attainment, and level of urbanicity. Gender was operationalized as self-reported sex assigned at birth.

### Country-Level Measures

#### Structural stigma

Scores for structural stigma toward sexual minorities in each country were based on a measure regarding population attitudes toward homosexuality and an index of discriminatory legislation and policies. Average country scores of population attitudes toward homosexuality, measured with the item “Gay men and lesbians should be free to live their own lives as they wish”, were derived from the 2012 European Social Survey (European Social Survey 2012). Data on country-level discriminatory and protective laws and policies were taken from the 2012 Europe Rainbow Index created by the International Lesbian, Gay, Bisexual, Trans and Intersex Association Europe (International Lesbian, Gay, Bisexual, Trans and Intersex Association Europe 2012). The measures were transformed into *z* scores and summed into a composite country-level structural stigma score, with higher scores indicating countries with higher levels of country-level structural stigma. Although the measure of country-level structural stigma was based on assessments during the year of the survey (i.e., 2012), this measure also likely reflects the relative rank-ordering of countries in terms of structural stigma present during the time in which the respondents attended school. Recent analyses from the US General Social Survey and by the Williams Institute indicate that the rank ordering of structural stigma climates for sexual minorities (by US state or by country, respectively) were relatively stable over a 30-year period in the US (results not shown, but available upon request) and over 36 years across 174 countries globally (Flores 2019).

#### Country-level prosperity

The gross domestic product at purchasing power parity per capita in 2012 was derived from the World Bank as a country-level covariate given its strong association with population life satisfaction as shown in previous studies (Boyce et al. 2010).

### Statistical Methods

In order to adjust for the nested structure of the data (by country), generalized linear mixed modeling (SPSS Statistics version 24; IBM Corporation, Armonk, NY, USA) was used to estimate cross-national and country-specific proportions and means. For the two gender groups separately, stepwise preparatory models and the final moderated serial mediation models used two-level random-slope modeling procedures in Mplus software (Muthén & Muthén, Los Angeles, CA, US; version 8) based on maximum likelihood with robust standard error estimates. The final models tested whether school bullying and subsequent adulthood victimization may explain the association between country-level structural stigma and adulthood life satisfaction, with identity openness as moderator of the country-level structural stigma to school bullying pathway. The final models were adjusted for nesting, respondents’ age, household income, and country-level prosperity. In all analyses, a significance level of *α* = 0.05 was used.

## Results

### Descriptive Statistics

Socio-demographic characteristics by gender group are summarized in Table 1. Table 2 presents the proportion of individuals reporting experiences of school bullying and being open about a LGB identity at school and the mean levels of adulthood victimization and adulthood life satisfaction by gender group. While sexual minority women were more open about their identity at school than sexual minority men, they were less likely to report school bullying but more likely to report adulthood victimization, compared with sexual minority men.

**Table 1:** Socio-demographic characteristics by gender group

	Total (*n* = 55,263)	Sexual minority women (*n* = 12,284)	Sexual minority men (*n* = 42,979)	*p* value
Age				<0.001
18–29, % (*n*)	53 (29,084)	72 (8799)	47 (20,285)	
30–39, % (*n*)	23 (12,916)	18 (2186)	25 (10,727)	
40–49, % (*n*)	16 (8728)	8 (930)	18 (7798)	
50–59, % (*n*)	6 (3454)	2 (284)	7 (3170)	
60+, % (*n*)	2 (1084)	1 (85)	2 (999)	
Sexual identity				<0.001
Lesbian/gay, % (*n*)	85 (46,823)	68 (8352)	90 (38,471)	
Bisexual, % (*n*)	15 (8440)	32 (3932)	10 (4508)	
Other, % (*n*)	0 (0)	0 (0)	0 (0)	
Ethnic minority				0.227
Self-identified ethnic minority, % (*n*)	4 (2412)	4 (512)	4 (1900)	
Education				<0.001
Achieved university level, % (*n*)	52 (28,833)	50 (6195)	53 (22,638)	
Household income				<0.001
Under first quartile, % (*n*)	28 (15,593)	34 (4197)	27 (11,396)	
Between first quartile and median, % (*n*)	25 (13,949)	26 (3216)	25 (10,733)	
Between median and third quartile, % (*n*)	22 (12,121)	22 (2641)	22 (9480)	
Above third quartile, % (*n*)	25 (13,600)	18 (2230)	27 (11,370)	
Urbanicity				0.013
Living in urban area, % (*n*)	89 (48,930)	89 (10,954)	88 (37,976)

**Table 2:** Proportions of school-based and adulthood victimization and the adjusted level of mean adulthood life satisfaction, by gender group

	Total (*n* = 55,263)	Sexual minority women (*n* = 12,284)	Sexual minority men (*n* = 42,979)	*p* value
School bullying				
Often/Always, % [95% CI]	35 [0.35, 0.36]	23 [0.23, 0.24]	38 [0.38, 0.39]	<0.001
Identity openness at school				
Often/Always, % [95% CI]	15 [0.14, 0.15]	20 [0.20, 0.20]	13 [0.12, 0.13]	<0.001
Adulthood victimization				
Experienced any victimization in past year, % [95% CI]	13 [0.13, 0.13]	13 [0.13, 0.13]	13 [0.12, 0.13]	0.008
Adulthood life satisfaction				
1–10, *M* [95% CI]	6.6 [6.61, 6.65]	6.7 [6.64, 6.71]	6.6 [6.59, 6.63]	0.013

Proportions were adjusted for the nested structure (by country) of the data

### Hypothesis 1: Association Between Country-Level Structural Stigma and School Bullying

In connection with the study’s first hypothesis, country-level structural stigma was weakly and negatively associated with school bullying among sexual minority women, but it was not significantly associated with school bullying among sexual minority men (Table 3).

**Table 3:** Estimates of intermediate steps to examine associations between country-level structural stigma, school-based and adulthood experiences, and adulthood life satisfaction among sexual minority women and men

	Multilevel model estimates					
	Sexual minority women (*n* = 12,284)			Sexual minority men (*n* = 42,979)		
	*b*	95% CI	*p*	*b*	95% CI	*p*
Level 2: Country-level estimates						
Total effects of country-level structural stigma on school-based experiences						
Country-level structural stigma on school bullying	–0.119	−0.232, −0.007	0.037	−0.006	−0.129, 0.117	0.925
Country-level structural stigma on identity openness at school	−0.281	−0.455, −0.107	0.002	−0.356	−0.491, −0.222	<0.001
Total effects of country-level structural stigma on adulthood experiences						
Country-level structural stigma on adulthood victimization	0.178	0.086, 0.270	<0.001	0.189	0.100, 0.278	<0.001
Country-level structural stigma on adulthood life satisfaction	−0.431	−0.541, −0.320	<0.001	−0.524	−0.635, −0.413	<0.001
Indirect effects of country-level structural stigma on adulthood life satisfaction						
Country-level structural stigma on school bullying on adulthood life satisfaction	0.011	0.002, 0.021	0.023	0.001	−0.013, 0.014	0.928
Country-level structural stigma on adulthood victimization on adulthood life satisfaction	−0.011	−0.016, −0.005	<0.001	−0.013	−0.020, −0.006	<0.001
Level 1: Individual-level estimates						
Total effects of school bulling on adulthood experiences						
School bullying on adulthood victimization	0.734	0.601, 0.868	<0.001	0.810	0.738, 0.882	<0.001
School bullying on adulthood life satisfaction	−0.483	−0.588, −0.377	<0.001	−0.447	−0.503, −0.390	<0.001
Total effect of adulthood victimization on adulthood life satisfaction						
Adulthood victimization on adulthood life satisfaction	−0.570	−0.673, −0.467	<0.001	−0.700	−0.793, −0.606	<0.001
Indirect effect of school bullying on adulthood life satisfaction						
School bullying on adulthood victimization on adulthood life satisfaction	−0.039	−0.052, −0.025	<0.001	−0.041	−0.051, −0.031	<0.001

Estimates were adjusted for the nested structure (by country) of the data

### Hypothesis 2: Identity Openness as Moderator of the Association Between Country-Level Structural Stigma and School Bullying

During preparatory tests leading up to the moderation analyses regarding the second hypothesis, country-level structural stigma was found to be negatively associated with identity openness at school among both sexual minority women and men (Table 3). Identity openness at school was significantly and positively associated with school bullying among both sexual minority women (*b* = 0.48, *p* < 0.001) and men (*b* = 0.26, *p* = 0.014).

**Fig. 1:**
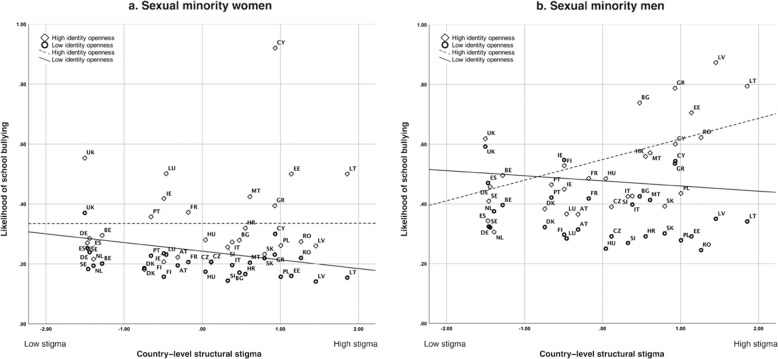
Cross-level moderation by identity openness of the association between country-level structural stigma and the likelihood of school bullying among sexual minority women (**a**) and sexual minority men (**b**). Note. Depicting proportions of school bullying adjusted for the nested structure (by country) and individual age. AT Austria, BE Belgium, BG Bulgaria, CY Cyprus, CZ Czech Republic, DE Germany, DK Denmark, EE Estonia, EL Greece, ES Spain, FI Finland, FR France, HU Hungary, HR Croatia, IE Ireland, IT Italy, LT Lithuania, LU Luxembourg, LV Latvia, MT Malta, NL Netherlands, PL Poland, PT Portugal, RO Romania, SE Sweden, SK Slovakia, SI Slovenia, UK United Kingdom

Regarding the second hypothesis, differences in the likelihood of school bullying between those who were open about their sexual identity at school and those not open were larger among sexual women and men living in higher-stigma countries compared to those living in lower-stigma countries (moderating effects: *b* = 0.15 [*p* = 0.003] and *b* = 0.31 [*p* = 0.003], respectively; Fig. 1). These results show that the association between higher levels of country-level structural stigma and a lower risk of school bullying was restricted to those sexual minority women who were not open about their identity at school (*b* = −0.14, *p* = 0.016; vs. those open, *p* = 0.859). Among sexual minority men, the association between higher levels of country-level structural stigma and a higher risk of school bullying was restricted to those sexual minority men who were open about their identity at school (*b* = 0.29, *p* = 0.012; vs. those not open, *p* = 0.785).

### Hypothesis 3: Indirect Effects of School Bullying on Adulthood Life Satisfaction

During preparatory tests for the third hypothesis, school bullying was found to be significantly and positively associated with adulthood victimization and both school bullying and adulthood victimization were significantly and negatively associated with adulthood life satisfaction among both sexual minority women and men (Table 3).

Regarding the third hypothesis, a higher risk of adulthood victimization did significantly explain a part of the association between experiences of school bullying and lower adulthood life satisfaction among both sexual minority women and men (Table 3). In models with adulthood victimization as mediator of the association between school bullying and adulthood life satisfaction, significant direct negative effects of school bullying on adulthood life satisfaction remained (sexual minority women: *b* = −0.44 [*p* < 0.001]; sexual minority men: *b* = −0.40 [*p* < 0.001]).

### Hypothesis 4: Integrated Models to Explore Indirect Effects of Country-Level Structural Stigma on Adulthood Life Satisfaction

During preparatory tests for the fourth hypothesis, sexual minority women and men living in countries with higher levels of structural stigma were found to report lower levels of adulthood life satisfaction than sexual minorities living in countries with lower levels of structural stigma (Table 3). Country-level structural stigma was significantly and positively associated with adulthood victimization among sexual minority women and men (Table 3). A higher risk of adulthood victimization was found to significantly explain a part of the association between higher levels of country-level structural stigma and lower adulthood life satisfaction among both sexual minority women and men (Table 3), while significant direct effects of country-level structural stigma on adulthood life satisfaction remained (sexual minority women: *b* = −0.43 [*p* < 0.001]; sexual minority men: *b* = −0.52 [*p* < 0.001]).

**Fig. 2:**
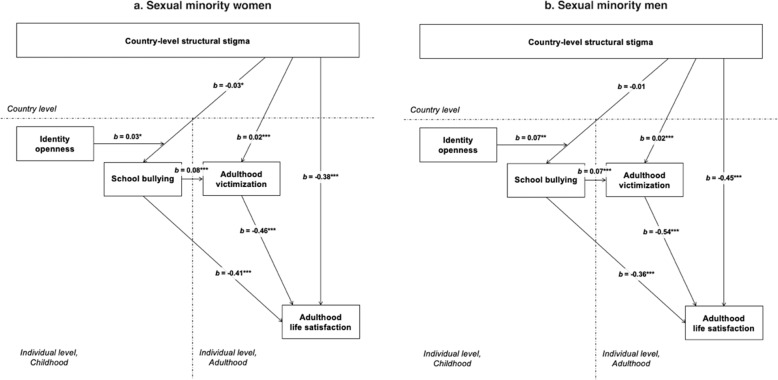
A model of cross-level moderation by identity openness of the mediation pathway of country-level structural stigma on adulthood life satisfaction through school bullying, with adulthood victimization as mediator of the pathway between school bullying and adulthood life satisfaction, and with school bullying and adulthood victimization as mediators of the pathway between country-level structural stigma and adulthood life satisfaction among sexual minority women (**a**) and sexual minority men (**b**). Note. Estimates were adjusted for nesting, age, household income, and country-level prosperity. *p < 0.05; **p < 0.01; ***p < 0.001

Regarding the fourth hypothesis, among sexual minority women who were not open about their identity at school, school bullying did weakly but significantly and positively explain the association between higher country-level structural stigma and lower adulthood life satisfaction (indirect effect: *b* = 0.01, *p* = 0.012) and, in parallel, between higher country-level structural stigma and lower adulthood life satisfaction through a higher risk of adulthood victimization (serial indirect effect: *b* = 0.01, *p* = 0.029; Fig. 2). In analyses further stratified by identity openness, these positive indirect effects accounted for approximately 2.6 and 0.3% of the otherwise negative association between country-level structural stigma and life satisfaction, respectively, among sexual minority women who were not open about their identity at school. No such indirect effects were found among sexual minority women who were open about their identity at school. Among sexual minority men who were open about their identity at school, school bullying did significantly and negatively explain the association between higher country-level structural stigma and lower adulthood life satisfaction (indirect effect: *b* = −0.02, *p* = 0.014) and, in parallel, between higher country-level structural stigma and lower adulthood life satisfaction through a higher risk of adulthood victimization (serial indirect effect: *b* = −0.01, *p* = 0.016; Fig. 2). In analyses further stratified by identity openness, these negative indirect effects accounted for approximately 9.7 and 1.4% of the negative association between country-level structural stigma and life satisfaction, respectively, among sexual minority men who were open about their identity at school. No such indirect effects were found among sexual minority men who were not open about their identity at school.

### Additional Analyses

To examine the robustness of the study results, sensitivity analyses with alternative variable coding were run to test the degree to which the dichotomizations of the school bullying, identity openness, and adulthood victimization variables might have affected the estimates of the indirect effects (Table 4). The alternative coding for both school bullying and identity openness at school included none vs. any of those experiences and, for adulthood victimization, a past-year frequency scale. The results from the moderated mediation analyses used to test Hypothesis 4 with this alternate coding remained similar to those analyses that used the original coding among sexual minority women who were not open about their identity at school. This was also true among sexual minority men who were open about their identity at school, except for the alternative coding of school bullying. These findings were consistent with the analyses using alternative variable coding to test Hypotheses 1–3. Specifically, with the alternative coding for school bullying, identity openness at school no longer significantly moderated the association between country-level structural stigma and school bullying among sexual minority men who were open at school (*p* = 0.077). Among sexual minority women who were open about their identity at school and among sexual minority men who were not open, indirect and serial indirect effects remained non-significant.

**Table 4:** Sensitivity analyses for different coding alternatives in bivariate, moderation, mediation, and moderated mediation models of country-level structural stigma, school-based and adulthood experiences, and adulthood life satisfaction among sexual minority women and men

	Multilevel model estimates					
	Sexual minority women (*n* = 12,284)			Sexual minority men (*n* = 42,979)		
	*b*	95% CI	*p*	*b*	95% CI	*p*
Hypothesis 1: Associations between country-level structural stigma and school bullying						
Original coding	−0.119	−0.232, −0.007	0.037	−0.006	−0.129, 0.117	0.925
Alternative coding of school bullying	−0.122	−0.202, −0.042	0.003	−0.099	−0.145, 0.087	0.623
Hypothesis 2: Moderation models of the association between country-level structural stigma and school bullying, moderated by identity openness at school	Estimates plotted at identity openness at school = low			Estimates plotted at identity openness at school = high		
Original coding	−0.136	−0.247, −0.025	0.016	0.287	0.063, 0.511	0.012
Alternative coding of school bullying	−0.100	−0.249, −0.031	0.005	0.285	−0.002, 0.572	0.051
Alternative coding of identity openness at school	−0.171	−0.266, −0.076	<0.001	0.164	0.017, 0.311	0.028
Hypothesis 3: Mediation models of the association between school bullying on adulthood life satisfaction through adulthood victimization						
Original coding	−0.039	−0.052, −0.025	<0.001	−0.041	−0.051, −0.031	<0.001
Alternative coding of school bullying	−0.035	−0.047, −0.023	<0.001	−0.038	−0.048, −0.028	<0.001
Alternative coding of adulthood victimization	−0.035	−0.049, −0.021	<0.001	−0.048	−0.059, −0.038	<0.001
Hypothesis 4: Moderated mediation models of the association between country-level structural stigma and adulthood life satisfaction through school bullying and subsequently adulthood victimization, moderated by identity openness at school	Estimates plotted at identity openness at school = low			Estimates plotted at identity openness at school = high		
Indirect effect through school bullying						
Original coding	0.010	0.002, 0.018	0.012	−0.024	−0.043, −0.005	0.014
Alternative coding of school bullying	0.004	0.002, 0.007	0.003	−0.006	−0.014, 0.004	0.099
Alternative coding of identity openness at school	0.012	0.006, 0.017	<0.001	−0.014	−0.027, −0.001	0.039
Alternative coding of adulthood victimization	0.010	0.002, 0.018	0.010	−0.023	−0.042, −0.005	0.012
Serial indirect effect through school bullying and adulthood victimization						
Original coding	0.001	0.001, 0.002	0.029	−0.002	−0.004, −0.001	0.016
Alternative coding of school bullying	0.001	0.001, 0.001	0.013	−0.001	−0.002, −0.000	0.073
Alternative coding of identity openness at school	0.001	0.001, 0.002	0.003	−0.001	−0.003, −0.001	0.038
Alternative coding of adulthood victimization	0.001	0.001, 0.001	0.027	−0.003	−0.005, −0.001	0.012

Estimates regarding Hypothesis 1–3 were adjusted for the nested structure (by country) of the data. Estimates regarding Hypothesis 4 were adjusted for nesting, age, household income, and country-level prosperity. Original coding for both school bullying and identity openness at school included never and rarely (= 0) vs. often and always (= 1), whereas alternative coding included never (=0) vs. any (= 1) of these experiences. Adulthood victimization was originally coded as none (= 0) vs. any (= 1) past-year experiences, while alternative coding was based on a past-year frequency scale

## Discussion

While structurally stigmatizing climates have been suggested to hamper sexual minorities’ wellbeing throughout their lives, most studies to date have only estimated associations between structural stigma, victimization experiences, and wellbeing in either childhood or adulthood in isolation (Hatzenbuehler 2017). Based on a large sample of sexual minorities living across widely varying structural climates, this study reports a gender-stratified life course exploration of whether exposure to structural stigma is associated with sexual minority adults’ life satisfaction through stigma-based experiences in both childhood (i.e., school bullying and identity openness at school) and adulthood (i.e., adulthood victimization). In this study, data from sexual minorities across 28 European countries permitted investigation of the life course correlates of structural stigma. The current study represents a comprehensive examination of the association between structural stigma toward sexual minorities and school bullying, with implications for adulthood life satisfaction.

This study found that many sexual minority adults across EU member states have been exposed to sexual identity-targeted school bullying. The association between country-level structural stigma toward sexual minorities (i.e., negative population attitudes and cultural norms, discriminatory legislation and policies, and unequal rights) and school bullying was dependent on whether or not sexual minorities were open about their lesbian, gay, or bisexual identity at school. Although sexual minority men reported an overall higher likelihood of exposure to school bullying, a similar pattern was found across genders regarding the association between country-level structural stigma and school bullying: higher rates of school bullying were found among those sexual minorities living in higher-stigma countries when they were open about their identity at school compared with those not open about their identity. More specifically, the negative association between higher levels of country-level structural stigma and a lower risk of school bullying was restricted to those sexual minority women who were *not open* about their lesbian or bisexual identity at school. Furthermore, only sexual minority men who were *open* about their gay or bisexual identity at school reported higher levels of school bullying when living in countries with higher levels of structural stigma.

Exposure to school bullying during childhood was associated with lower adulthood life satisfaction. In terms of life course hypotheses, this study found some indication that the negative association between country-level structural stigma and sexual minorities’ adulthood life satisfaction seemed to be indirectly linked through childhood school-based experiences and subsequent experiences of victimization in adulthood. However, these indirect effects only accounted for small proportions of the association between country-level structural stigma and life satisfaction. Nonetheless, the results indicate that some sexual minorities in higher-stigma countries may use visibility management strategies regarding their emerging identities during school years to mitigate the risk of school bullying, which might hold benefits for wellbeing in adult life. However, these results should be interpreted with caution as they do not suggest that identity concealment would be beneficial to sexual minorities in other settings beyond the school environment, during other developmental periods, or in other domains of sexual minorities’ lives. In fact, a large body of research demonstrates the psychological toll of identity concealment (Pachankis 2007). While concealment may be culturally desired (Schrimshaw et al. 2018) or even privileged in some cultures (Massad 2002), sexual identity concealment has also been linked to increased levels of distress through several mentally taxing processes (Pachankis 2007). These processes include continuous visibility management efforts, the anxious anticipation of rejection by others, and the threat of discovery (Pachankis 2007). Sexual identity concealment may also hamper the development of a positive sexual identity and authentic self and any access to a global sexual and gender minority movement. At the same time, alignment with a singular sexual and gender minority movement is not necessarily associated with positive outcomes for all sexual and gender minority individuals, even if it has the ability to advance sexual and gender minority rights. These multilevel considerations no doubt shape the positive and negative consequences of the closet.

The study findings shed new light on the mechanisms underlying the link between country-level structural stigma and sexual minorities’ adulthood wellbeing by taking into account childhood and subsequent adulthood experiences of victimization. Previous studies have focused either on structural stigma, childhood experiences, and childhood wellbeing (e.g., Saewyc et al. 2014) or on structural stigma, adulthood experiences, and adulthood wellbeing in isolation (e.g., Pachankis and Bränström 2018). However, this study suggests that country-level structural stigma might shape childhood experiences with implications for adulthood experiences and life satisfaction. Specifically, the results indicate that structural stigma is not only associated with wellbeing of sexual minority adults through *contemporaneous* adulthood victimization, but also through *historical* experiences that might increase the likelihood of stressful experiences during childhood and adulthood. This is of particular importance given the results from earlier studies suggesting that peer victimization at a young age may lead to subsequent anticipated rejection in adulthood through lower self-confidence and less assertiveness in social interactions, which in turn could make one vulnerable to accumulated social stress and compromise wellbeing (Earnshaw et al. 2016).

Whereas these results seem to suggest that the mechanisms related to historical correlates of country-level structural stigma may explain more of its adulthood sequelae among sexual minority men than among sexual minority women, it remains unknown whether this may be due to, for instance, a higher risk of school bullying, an increased frequency of school bullying over time, or a higher likelihood of being targeted due to gender non-conforming behavior among sexual minority boys. In line with the study results, previous research has shown that sexual minority boys experience more frequent stigma-based school bullying than sexual minority girls (Chesir-Teran and Hughes 2009). The frequency of stigma-based school bullying among sexual minority boys may increase over time, while among sexual minority girls the frequency has been shown to progressively decrease (Poteat et al. 2012). Several studies have also reported a higher risk of bullying based on gender non-conforming behaviors among sexual minority boys (D’Augelli et al. 2006), although less is known about how sexual minority girls may experience stigma-based school bullying distinctively (Poteat and Russell 2013), particularly in relation to gender differences in bullying severity, in its form, and in psychosocial adjustment to stigma-based bullying experiences. Research on gender differences in body weight-based bullying has found that boys and girls may be exposed to different forms of bullying, e.g., physical vs. emotional, respectively (Wang et al. 2010). Whether psychosocial adjustment in later life to stigma-based bullying is similar for sexual minority boys and girls, as one study on gender non-conformity suggests (Toomey et al. 2010), remains to be further investigated. Regarding the study’s hypothesis that sexual minority boys would have fewer opportunities to use identity non-disclosure at school in order to avoid sexual identity-based bullying in higher-stigma countries, no clear indications were found; identity non-disclosure at school was associated with lower risk for school bullying among sexual minority women in higher-stigma countries, while identity openness was associated with increased risk among sexual minority men in such countries. The overall pattern was similar across genders with a higher risk for school bullying among those sexual minorities open about their identity at school in higher-stigma countries. More research is needed to further unravel the potentially distinct ways in which historic and contemporaneous consequences of country-level structural stigma may comprise sexual minority wellbeing across genders, for instance, by looking at exposure to structural stigma before, during, and after the formation of a sexual identity.

The results should be interpreted in light of several limitations. First, this non-probability sample of participants cannot be regarded as representative of the sexual minority populations in each country. For instance, recruitment using local LGBT organizations and social networking web sites might lead to an overrepresentation of sexual minority individuals who are open about their sexual identities (Meyer and Wilson 2009). Second, in order to ensure accurate classification of respondents’ childhood structural stigma exposure and given the lack of information on migration routes, respondents who did not attend school in their country of residence were excluded from the study. This may have affected the results, as structural environments and past victimization may also be motivations for sexual minorities to migrate from and escape homophobic climates, which in turn might shape their life trajectories (Pachankis et al. 2016). Third, no information was available on sexual identity during childhood and adolescence. Country-level structural stigma may affect the formation and stability of a sexual identity (Ott et al. 2011), as the prevalence of minority sexual identities has been shown to vary as a function of country-level structural stigma among sexual minority men (Pachankis et al. 2017), with sexual identity stability potentially influencing the association between structural stigma and sexual identity-based school bullying. Fourth, the data showed a high degree of non-random missingness on our key study variables, but extensive imputation of data was deemed inappropriate. Because most of the variables were treated as mediating or outcome dependent variables, maximum likelihood estimations were only partially helpful and respondents with missing data on these variables were excluded from analyses. As the included sample generally reported more negative outcomes than the excluded group, this may have led to selection bias. Fifth, although some of the survey questions were of retrospective nature, the cross-sectional design did not allow for drawing causal inferences. Although common in bullying research (Collier et al. 2013b), retrospective reports of childhood experiences may be subject to recall bias. Sixth, the subjective measures of identity-targeted school bullying experiences relied on a self-interpretation that being lesbian, gay, or bisexual was the primary reason for the peer victimization. Such a subjective interpretation or recall is potentially influenced by structural stigma or induced by adverse adulthood experiences but would, hence, also lie along the suggested causal pathway between structural stigma and school bullying or adulthood life satisfaction, and not confound the results. Lastly, limited information was available on several other potential mediators of the association between structural stigma and sexual minority wellbeing. While well-established risk factors, such as internalized homophobia and lack of social support (e.g., Berg et al. 2013), were not assessed in the EU-LGBT survey, these risk factors have also been linked to childhood victimization (Bergeron et al. 2015). Therefore, these unobserved factors would not bias the estimates as they would lie along the hypothesized causal pathways from structural stigma to low life satisfaction.

## Conclusion

Although research has suggested that structurally stigmatizing environments may shape sexual minorities’ mental health across the life course, the ways in which exposure to structural stigma may give rise to stigma-based experiences in both childhood and adulthood to affect sexual minority adults’ life satisfaction has not been previously examined. Taking advantage of a large dataset of sexual minorities across 28 European countries, this study found that large proportions of sexual minorities experience school bullying both in higher- and lower-stigma countries in Europe. In higher-stigma settings, sexual minorities who are open about their sexual identity at school are more likely to experience school bullying. School bullying seems to be associated with lower adulthood life satisfaction, also when taking into account how adulthood experiences of victimization may explain this association. The findings suggest that, despite the established costs of concealment, some sexual minorities living in higher-stigma countries may nonetheless benefit from not being open about their sexual identity at school, by reducing their risk for school bullying and subsequent adverse adulthood experiences, providing one of the first indications that structural stigma is associated with sexual minority adults’ wellbeing not only through contemporaneous, but also historical, experiences of victimization.
